# Previous Abdominal Scars among Patients Undergoing Laparoscopic Cholecystectomy in a Tertiary Care Centre

**DOI:** 10.31729/jnma.8240

**Published:** 2023-08-31

**Authors:** Roshan Ghimire, Prashanta Pudasaini, Bidur Prasad Acharya, Yugal Limbu, Sujan Regmee

**Affiliations:** 1 Department of Gastrointestinal and General Surgery, Kathmandu Medical College and Teaching Hospital, Sinamangal, Kathmandu, Nepal

**Keywords:** *laparoscopic cholecystectomy*, *open surgery*, *prevalence*

## Abstract

**Introduction:**

Abdominal scars result from various open abdominal surgeries. Laparoscopic surgery in previous open abdominal surgery possesses various challenges to the surgeon like gaining access to the abdominal cavity, and difficulty in dissection due to dense adhesions from previous surgeries for various intraabdominal pathologies. This study aimed to find out the prevalence of previous abdominal scars among patients undergoing laparoscopic cholecystectomy in a tertiary care centre.

**Methods:**

A descriptive cross-sectional study was conducted among patients undergoing laparoscopic cholecystectomy in a tertiary care centre from 1 May 2022 to 30 April 2023 after taking ethical approval from the Institutional Review Committee. Palmer's point approach via Hassen open technique or direct optical entry was used for cases with previous abdominal scars to gain access to the abdominal cavity. Patients with symptomatic gallstone diseases were included in the study whereas patients with cholecystitis, pancreatitis, and previous cesarean scar were excluded. Convenience sampling method was used. The point estimate was calculated at a 95% Confidence Interval.

**Results:**

Among 160 patients undergoing laparoscopic cholecystectomy, previous abdominal scars was found in 40 (25%) patients.

**Conclusions:**

The prevalence of previous abdominal scars contributing to intraoperative and postoperative difficulties among patients undergoing laparoscopic cholecystectomy was found to be higher than in studies done in similar settings.

## INTRODUCTION

Laparoscopic cholecystectomy has become the procedure of choice for symptomatic gallstone disease due to its minimal invasiveness, less post-operative pain, earlier oral intake, less hospitalization, better cosmetic results and early return to work.^1^ Approximately 1-2% of asymptomatic gallstones will develop symptoms requiring cholecystectomy.^2^

Laparoscopic surgery in previous open abdominal surgery possesses challenges like gaining access to the abdominal cavity and difficult dissection due to dense adhesions. Adhesions are the most common driver of long-term morbidity after abdominal surgery^3^ Abdominal scars can contribute to intraoperative and post-operative difficulties among patients undergoing laparoscopic cholecystectomy.^4,5^

This study aimed to find out the prevalence of previous abdominal scars among patients undergoing laparoscopic cholecystectomy in a tertiary care centre.

## METHODS

A descriptive cross-sectional was conducted among patients undergoing laparoscopic cholecystectomy in Kathmandu Medical College and Teaching Hospital, Sinamangal, Kathmandu, Nepal from 1 May 2022 to 30 April 2023 after obtaining ethical approval from the Institutional Review Committee (Reference number: 2003202204). Informed consent was obtained from all patients for the utilization of their data for research purposes. All patients with symptomatic gallstone disease undergoing laparoscopic cholecystectomy during the study period were included in the study whereas patients with cholecystitis, pancreatitis, laparoscopic cholecystectomy as a part of other surgeries, previous cesarean scar, physical status with grading of American Society of Anesthesiologist 3 or more were excluded. Convenience sampling was done. The sample size was calculated using the following formula:


$$\begin{array}{ll} \text{n} & ={\text{Z}}^{2}\times \frac{\text{p}\times \text{q}}{{\text{e}}^{\text{2}}}\\ & =1.96^{2}\times \frac{0.50\times 0.50}{0.08^{2}}\\ & =150 \end{array}$$

Where,

n = minimum required sample size
Z = 1.96 at 95% Confidence Interval (CI)
p = prevalence taken as 50% for maximum sample size calculation
q = 1-p
e = margin of error, 8%

The calculated sample size was 150. However, 160 patients were included in the study.

Preoperative physical status was graded according to the American Society of Anaesthesiologists (ASA) guidelines, and all patients underwent ultrasonography, when required, other advanced imaging was done. A standard 3/4 ports laparoscopic cholecystectomy was performed. Raoul Palmer MD described Palmer's point as the area in the left upper quadrant 3 cm below the costal margin in the midclavicular line.^4^ Hasson described an open entry technique avoiding the insertion of needles or sharp trocars blindly.^4^ Another safe approach is direct optical entry through palmers point which allows visual inspection and adhesiolysis to allow subsequent insertion of an umbilical port under vision.^6^ Palmer's point approach was used for cases with midline scars from previous open surgeries for gaining access. The left upper quadrant was inspected for scars and the upper abdomen was palpated for hepatomegaly or splenomegaly. Open Hassen technique or direct optical entry was used to open Palmer's point. A 10 mm incision was made over the skin in Palmer's point. The abdomen was opened in layers.^6^

The layers of the abdominal wall seen at Palmer's point are skin, subcutaneous fat, external oblique aponeurosis, internal oblique aponeurosis,transversalis muscle fibres, (sometimes) extraperitoneal fat and peritoneum. Once the peritoneum was breached insufflation was commenced. After gaining access to Palmer's point, other ports were created under vision. Umbilical port and right subcostal ports were created and if necessary epigastric subxiphoid port was created as per the surgeon's confidence. Adhesiolysis was done under vision. A critical view of safety was achieved in most of the cases and if needed bailout procedures were considered.^6^ At the end of the operation, ports were closed and the skin was sutured. Preoperative demographic and clinical data, surgical procedure, pathological diagnosis and postoperative course were collected from maintained data and analyzed. Postoperative complications were graded according to Clavien-Dindo.^7^

Data were entered in Microsoft Excel 2016 and analysed using IBM SPSS Statistics version 20.0. The point estimate was calculated at a 95% Confidence Interval.

## RESULTS

Among 160 patients undergoing laparoscopic cholecystectomy, a previous abdominal scar was found in 40 (25%) (18.29-31.71, 95% CI) patients. The predominant age group undergoing surgery was 31-50 years with a female predominance with a male: female ratio of 1:3 (Table 1).

**Table 1:** Demographic details (n= 40).

Variables		n (%)
Age (years)	18-30	8 (20)
	31-50	18 (45)
	51-70	12 (30)
	>70	2 (5)
Sex	Male	10 (25)
	Female	30 (75)
	<18.5 (underweight)	3 (7.50)
Body mass index (BMI)		
	18.5-24.9 (normal)	7 (17.50)
	25-29.9 (preobese)	19 (47.50)
	>30 (obese)	11 (27.50)
Previous scar mark		Midline laparotomy	9 (22)
		Lanz	8 (21)
		Gridiron	5 (12.50)
		Low transverse	7 (17)
		Mini lap umbilical scar	10 (25)
		Right subcostal	1 (2.50).

Among 40 patients, 32 (80%) weregraded asgrade 1 accordingtoASA grading(Table 2).

**Table 2:** Grading according to ASA (n= 40).

Variables		n (%)
**ASA**	Grade 1	32 (80)
	Grade 2	8 (20)

Among 40 patients, 32 (80%) were graded as Grade 1 according to Clavein-Dindo grading and 8 (20%) were under Grade 2.

Among 40 patients with abdominal scar, 4 (10%) required intraoperative cholangiogram and 3 (7.5%) patients' scar was comverted to open. The reason for conversion was due to dense omental adhesion resulting in difficulty in port placement in 2 patients (5%) and type 1 Mirizzi syndrome in 1 patient (2.5%) (Table 3).

**Table 3:** Fate of patients with abdominal scar (n = 40).

Characteristics	Factors	n (%)
Conversion to open	Dense omental adhesion (difficult port placement) Type 1 Mirizzi syndrome	2 (5)
Subtotal	Inflamed gallbladder	1 (2.5)
cholecystectomy	Gallbladder perforation	1 (2.5)
(Reconstituting and Fenestrating) Inability to achieve CVS	Fibrotic contracted gallbladder	1 (2.5)
Need of intraoperative cholangiogram	Suspected right posterior sectoral duct injury	2 (5)
	Suspected CBD injury	2 (5)

## DISCUSSION

Among 160 patients undergoing laparoscopic cholecystectomy, a previous abdominal scar was found in 40 (25%) which is lower than 50% taken for maximum sample size calculation.

The predominant age group undergoing surgery was 31-50 years 18 (45%). In our study, majority of the patients were female 30 (75%). In a past study, 65% were female.^8^ In our study, adhesion was found in 2 (5%) patients whereas in a previous study, it was found that umbilical ashesion was found to be 1.6% and that adhesion is one of the commonly encountered problem.^9-11^ In a similar study, 13-1% had developed adhesion.^12^ In our study, 32 (80%) patients were graded as Grade 1 according to Clavein-Dindo grading system. In a previous study, 38 (55.07%) were graded as Grade 1.^13^

The major limitation of this study is that it is a singlecentred study and a small sample size was taken so the data cannot be generalised to a greater level. Also, since this is a descriptive cross-sectional study, analytical parameters could not be evaluated.

## CONCLUSIONS

The prevalence of previous abdominal scars among patients undergoing laparoscopic cholecystectomy was found to be lower than studies done in similar settings. Preoperative anticipation of difficulties during LC in patients with previous abdominal scars reduces the operative stress and prevents postoperative morbidity.
